# Great Expectations: The Implementation of Integrated Care and Its Contribution to Improved Outcomes for People with Chronic Conditions

**DOI:** 10.5334/ijic.2555

**Published:** 2016-12-31

**Authors:** Loraine Busetto

**Affiliations:** Tilburg University, NL

**Keywords:** integrated care, chronic conditions, evaluation, implementation, diabetes, geriatric conditions, workforce

## Abstract

There are great expectations regarding the potential contribution of integrated care interventions to improved outcomes, but so far the evidence is mixed. In this dissertation, we focussed on why, when and how some integrated care interventions contribute to improved outcomes, while others do not. To this purpose, we developed the COMIC Model for studying the **C**ontext, **O**utcomes and **M**echanisms of **I**ntegrated **C**are interventions. Evaluations that make use of the COMIC Model take into account the context in which an intervention is implemented and can thereby provide insights into why an intervention does (not) work and how the intervention and/or the context can be changed to achieve improved outcomes.

## Introduction

Integrated care is seen as one of the most promising approaches to providing appropriate care to people with (multiple) chronic conditions. There are great expectations regarding the outcomes integrated care is supposed to achieve, including improved quality of care and health outcomes, better patient experiences and increased cost efficiency. However, so far, findings have been mixed, with some studies indicating improved outcomes [1,2,3,4] and others pointing towards mixed evidence or no improvements [1,3,5,6,7,8,9,10]. In this dissertation,^1^ we aimed to understand when, why and how some integrated care interventions contribute to improved outcomes, while others do not. Specifically, we aimed to answer the research question: *How is integrated care implemented and to which outcomes does it contribute?*

**Figure 1:**
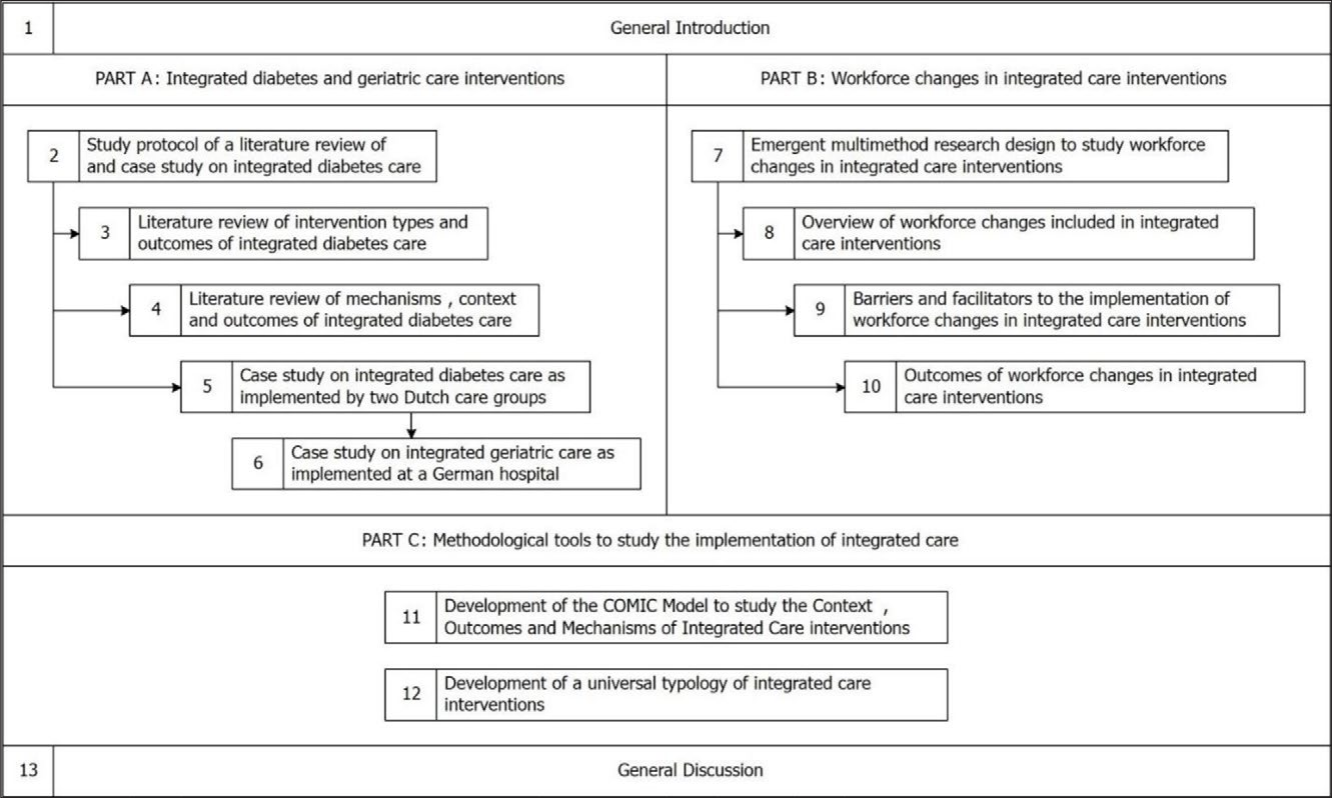
Overview of the studies included in the dissertation. Note: The studies are numbered according to the respective chapters of the dissertation in which they are presented, starting with a General Introduction in Chapter 1 and ending with a General Discussion in Chapter 13. Arrows indicate that studies are based on insights presented or methodologies developed in previous studies.

We approached this question from different angles. In Part A of the dissertation, we studied the implementation of integrated care interventions for two different (groups of) chronic conditions, namely type 2 diabetes and geriatric conditions. In Part B, we focussed on a specific aspect of integrated care, namely workforce changes, implemented as part of integrated care interventions. In Part C, we developed methodological tools to support comprehensive evaluations of when, why and how integrated care interventions can contribute to improved outcomes. This included the development of the COMIC Model for studying the **C**ontext, **O**utcomes and **M**echanisms of **I**ntegrated **C**are interventions. The different studies included in this dissertation are summarised in Figure 1. In this summary, we only focus on the COMIC Model.

## Main findings

Comprehensive evaluations of integrated care interventions that aim to answer the when, why and how of successful outcomes must focus on the interplay between mechanisms, context and outcomes. The importance of this interplay has been described most appropriately in the Context-Mechanisms-Outcomes (CMO) Model, which postulates that interventions only have successful outcomes when they introduce appropriate mechanisms in the appropriate social and cultural contexts [11]. However, there is no consensus on the definition and operationalisation of what exactly is meant by the concepts “context”, “mechanisms” and “outcomes” [12,13]. This is problematic for the consistent application of the model to the collection and analysis of empirical data, as well as the comparison of findings across studies.

**Figure 2:**
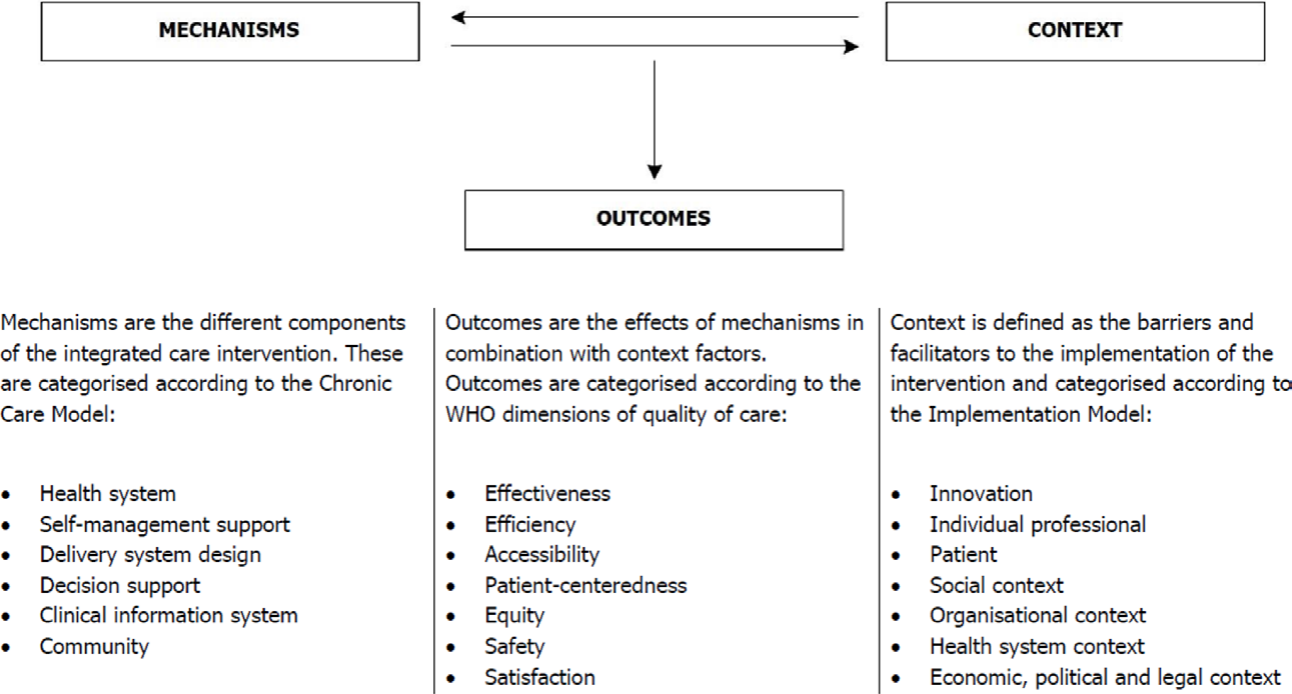
COMIC Model to study the Context, Outcomes and Mechanisms of Integrated Care interventions.

In response to these challenges, we developed a model that provides definitions and operationalisations of these elements, as well as a visualisation of the interplay between these elements. The COMIC Model (Figure 2) to study the **C**ontext, **O**utcomes and **M**echanisms of **I**ntegrated **C**are interventions assumes that an intervention is introduced using certain mechanisms, which are met with certain context factors, which combined, contribute to certain outcomes. Mechanisms are defined as the different components of an integrated care intervention and categorised according to the Chronic Care Model [14]. Context is defined as the setting in which mechanisms are brought into practice, described by barriers and facilitators and categorised according to the Implementation Model [15]. Outcomes are defined as effects triggered by mechanism and context and categorised by the World Health Organization’s dimensions of quality of care [16,17].

Our research has shown that the COMIC Model makes it possible to comprehensively analyse mechanisms, context and outcomes within a given case, to visualise the relationships between the mechanisms, context and outcomes within a given case, and to compare several cases to each other in a systematic way that adds value to the analysis.

## Recommendations

The good news is that integrated care interventions have been shown to be able to contribute to improved outcomes. Optimism seems to be warranted, as do further investments (financial or intellectual) in this area. However, negative outcomes and no improvements have been found as well and it seems naïve to think that if only we found the perfect intervention, the occurrence of negative outcomes could be prevented. Instead, it is more likely that any complex intervention will contribute to positive as well as negative outcomes, and the main question should therefore be how to curtail the negative and boost the positive ones. This entails that we see outcomes not as endpoints of an evaluation, but indicators of how an intervention can be improved and opportunities to actually do so. This observation is in line with a more general call for intervention improvement rather than the “accreditation” or “freezing” of the intervention itself as well as the way it is implemented [18,19]. As Chambers et al. have argued, there is no reason why health services research should not use continuous improvement cycles as the ones used for software development, which aim for improved versions 2.0 and higher [19]. Comprehensive evaluations of the initial implementation of interventions should be used to collect useable information on which areas need to be improved and in which ways this can be realised. This would call for an increased focus on improving the “fit” between the context and the intervention [19], underscoring the necessity of not only focusing on the intervention to be implemented, but also on making sure that the circumstances are right for the intervention to be carried out. This also holds true in the long run after the initial implementation and evaluation have taken place. We expect that the COMIC Model can assist researchers and practitioners in finding current mismatches between context and mechanisms and thereby point towards solutions that can contribute to improved outcomes in the future.

## Conclusions

This dissertation has investigated the question of how integrated care is implemented and to which outcomes it contributes. Of course, an improved understanding of the implementation of integrated care is not an aim in itself, but stems from the desire to implement better interventions, and to implement them better, in order to achieve better outcomes, and to do so more consistently. We expect that the insights from this dissertation, and in particular the COMIC Model to study the **C**ontext, **O**utcomes and **M**echanisms of **I**ntegrated **Ca**re interventions, will support future comprehensive evaluations of integrated care interventions. By focussing on the implementation of an intervention, including which type of intervention was implemented, how the setting in which the intervention was implemented affected its implementation, and which outcomes were achieved, these evaluations are expected to contribute to improved outcomes for people with or at risk of chronic disease. This is not a ready-made solution, but an instrument to be put in the hands of researchers, policy-makers, practitioners and patients.

## Footnotes

The research conducted within the scope of the dissertation was part of Project INTEGRATE and therefore co-subsidised by the European Union.

## Author Information

Dr. Loraine Busetto is a researcher in the field of integrated care. She holds a Ph.D. from Tilburg University and a M.Sc. from the University of Twente, the Netherlands.

## Published articles by the author relevant to this PhD thesis

**Busetto**, L, Luijkx, KG and Vrijhoef, HJM. Development of the COMIC Model for the comprehensive evaluation of integrated care interventions. International Journal of Care Coordination. 2016; 19(1–2), 47–58. DOI: http://dx.doi.org/10.1177/2053434516661700. http://icp.sagepub.com/content/early/2016/07/27/2053434516661700.abstract [3 Nov 2016].

**Busetto**, L, Luijkx, KG, Calciolari, S, González Ortiz, LG and Vrijhoef, HJM. Outcomes of integrated chronic care interventions including workforce changes: an expert questionnaire and literature review. European Journal for Person Centered Healthcare. 2016. Accepted for publication.

**Busetto**, L, Luijkx, KG, Elissen, AMJ and Vrijhoef, HJM. Context, mechanisms and outcomes of integrated care for diabetes mellitus type 2: a systematic review. BMC Health Services Research. 2015; 16: 18. DOI: http://dx.doi.org/10.1186/s12913-015-1231-3. http://bmchealthservres.biomedcentral.com/articles/10.1186/s12913-015-1231-3 [3 Nov 2016].

**Busetto**, L, Luijkx, KG, Elissen, AMJ and Vrijhoef, HJM. Intervention types and outcomes of integrated care for diabetes mellitus type 2: a systematic review. Journal of Evaluation in Clinical Practice. 2016; 22(3), 299–310. DOI: http://dx.doi.org/10.1111/jep.12478. http://onlinelibrary.wiley.com/doi/10.1111/jep.12478/abstract [3 Nov 2016].

**Busetto**, L, Luijkx, KG, Huizing, A and Vrijhoef, HJM. Implementation of integrated care for diabetes mellitus type 2 by two Dutch care groups: a case study. BMC Family Practice. 2015; 16: 105. DOI: http://dx.doi.org/10.1186/s12875-015-0320-z. https://bmcfampract.biomedcentral.com/articles/10.1186/s12875-015-0320-z [3 Nov 2016].

**Busetto**, L, Luijkx, KG and Vrijhoef, HJM. Implementation of Integrated Care for Type 2 Diabetes: A Protocol for Mixed Methods Research. International Journal of Integrated Care. 2014; 14 (Oct–Dec), e033. DOI: http://dx.doi.org/10.5334/ijic.1516. http://www.ijic.org/index.php/ijic/article/view/1516/2799 [3 Nov 2016].
